# Likelihood Inference of Non-Constant Diversification Rates with Incomplete Taxon Sampling

**DOI:** 10.1371/journal.pone.0084184

**Published:** 2014-01-06

**Authors:** Sebastian Höhna

**Affiliations:** Department of Mathematics, Stockholm University, Stockholm, Sweden; British Columbia Centre for Excellence in HIV/AIDS, Canada

## Abstract

Large-scale phylogenies provide a valuable source to study background diversification rates and investigate if the rates have changed over time. Unfortunately most large-scale, dated phylogenies are sparsely sampled (fewer than 5% of the described species) and taxon sampling is not uniform. Instead, taxa are frequently sampled to obtain at least one representative per subgroup (e.g. family) and thus to maximize diversity (diversified sampling). So far, such complications have been ignored, potentially biasing the conclusions that have been reached. In this study I derive the likelihood of a birth-death process with non-constant (time-dependent) diversification rates and diversified taxon sampling. Using simulations I test if the true parameters and the sampling method can be recovered when the trees are small or medium sized (fewer than 200 taxa). The results show that the diversification rates can be inferred and the estimates are unbiased for large trees but are biased for small trees (fewer than 50 taxa). Furthermore, model selection by means of Akaike's Information Criterion favors the true model if the true rates differ sufficiently from alternative models (e.g. the birth-death model is recovered if the extinction rate is large and compared to a pure-birth model). Finally, I applied six different diversification rate models – ranging from a constant-rate pure birth process to a decreasing speciation rate birth-death process but excluding any rate shift models – on three large-scale empirical phylogenies (ants, mammals and snakes with respectively 149, 164 and 41 sampled species). All three phylogenies were constructed by diversified taxon sampling, as stated by the authors. However only the snake phylogeny supported diversified taxon sampling. Moreover, a parametric bootstrap test revealed that none of the tested models provided a good fit to the observed data. The model assumptions, such as homogeneous rates across species or no rate shifts, appear to be violated.

## Introduction

Patterns of biodiversity reflected in phylogenetic estimates indicate that (1) rates of diversification are not constant over time or across the tree and (2) taxonomic sampling is both incomplete and non-random. Furthermore, knowing the sampling strategy is crucial for unbiased estimation of diversification rates [1]–[3]. Researchers sample species in a manner that is not uniform. Instead, taxa are often selected so that the diversity is maximized, e.g. sampling at least one species per family [4], [5]. This strategy is called *diversified* sampling [2].

Several extensions have been proposed to model non-constant diversification rates [6]–[9] but the combination of incompletely sampled phylogenies and non-constant rates has attracted less attention. It is well known how to accommodate uniform taxon sampling (also called random sampling), where every taxon has the same probability to be included in the dataset, in inference based on the birth-death process [6], [10]. The birth-death process with uniform taxon sampling has been extended to time-dependent rates [8] and diversity-dependent rates [11]. Diversified taxon sampling has only been considered in the context of constant rates [2] and, to my knowledge, the corresponding likelihood functions for non-constant rates have not been available previously.

In the present paper I derive the likelihood function for the birth-death process with diversified taxon sampling and time-dependent diversification rates. Thus, the diversification rates may be defined as any function that only depends on the variable time e.g. an exponentially decaying speciation rate. The focus of the paper lies on frequentist inference of the parameters of the birth-death process by means of Maximum Likelihood Estimation (MLE) and model selection by means of the Akaike Information Criterion (AIC) and Bayesian Information Criterion (BIC).

It has been claimed that MLEs of speciation and extinction rates are unbiased if the correct model is used for the inference [8], [12] although the MLE is not always an unbiased estimator [13]. I will investigate by means of simulations if the MLE is biased.

Selection of different birth-death models using the AIC score has been widely applied and used to reject constant-rate models [9], [14]–[16] although Rabosky [7] showed that the AIC score may be misleading. Therefore I investigate the power of the AIC and BIC scores to recover the true model.

Besides the use in Maximum Likelihood inference as in this study, the derived likelihood functions are also important for Bayesian phylogenetic inference. The birth-death process assigns a probability distribution on phylogenetic trees and divergence times. Thus, the birth-death process is frequently used in Bayesian phylogenetic inference as a prior probability distribution [10], [17]–[20]. This prior probability distribution influences divergence times estimates and more realistic birth-death models improve the estimation of divergence times and rates of molecular evolution [21]–[23].

Eventually, I applied six different diversification rate models on three large-scale phylogenies: an ant phylogeny [24], a mammal phylogeny [4] and a snake phylogeny [25]. These three phylogenies have in common that (1) they are relatively large (149, 164 and 41 sampled taxa), (2) are sparsely sampled (fewer than 5% of the known species included) and (3) taxa were included as representatives of different clades (diversified sampling). I conclude by investigating the adequacy of the used models (model fit) and discuss model shortcomings and potential model improvements.

## Materials and Methods

### The birth-death process with non-constant diversification rates and diversified taxon sampling

Following the notation of Nee et al. [6], I define the birth-death process with non-constant rates and diversified sampling for rooted, strictly bifurcating trees. At any given point in time 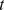, the number of living species is denoted by 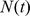. The number of species 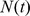 is modeled by a Markovian birth and death process with individual birth and death rates given by 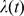 and 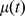.

**Figure 1:**
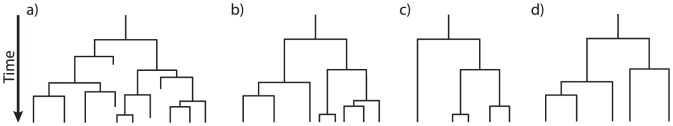
Sketches of a tree produced by a birth-death process. The process starts with a single lineage at the origin. At each speciation event the ancestral lineage is replaced by two descendant lineages. At an extinction event the lineage simply terminates. a) A complete tree including the extinct lineages. b) The reconstructed tree of tree a) without the extinct lineages. c) A randomly sampled tree of the same reconstructed tree. Every taxa had the same probability 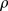 to be sampled. d) A five-taxon tree where taxa are selected to maximize diversity (diversified sampling).

**Figure 2:**
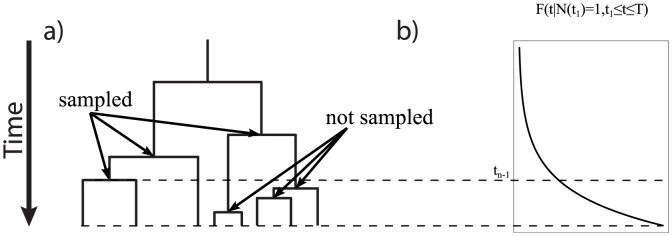
Illustration of diversified sampling and the probability density. a) A reconstructed tree where three speciation events are sampled and last three speciation events are not. b) The cumulative probability density of the time of a speciation event. All non-sampled speciation events occurred some time after the dashed line (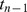) and thus have the probability 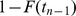.

The process starts with two species at time 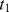 – which is the time of the most recent common ancestor and therefore 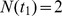. For each species, a speciation or an extinction event occurs randomly with rates 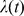 and 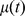 respectively. At a speciation event, the ancestral species splits into two descendant species and at an extinction event the species dies. The events happen instantaneously and speciation and extinction rates are equal for all species. At the present time 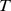 the process is stopped. An example of this process is illustrated in Figure 1.a. Then, the extinct lineages are removed because only the extant taxa are observed (see Figure 1.b). Let 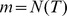 denote the number of extant species at time 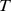 in the complete tree (see Figure 1.b) out of which 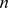 species are sampled. Under uniform taxon sampling, every extant species at the current time 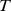 has the same probability 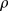 to be sampled and included in the reconstructed tree (see Figure 1.c). Under diversified sampling, all species that arose from the last 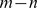 speciation events are removed (see [2] for a more detailed description). The process is illustrated in Figure 1 and Figure 2.

Starting with one species at time 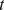 (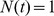) there are three key probability functions: the probability of survival (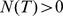), exactly 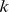 species (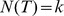) and exactly one species at time 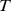 (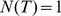) given by [6], [26]

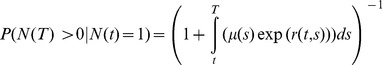
(1)

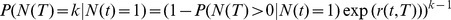


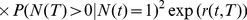
(2)

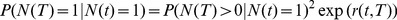
(3)where 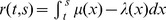. Equation (1) is derived from Equation (24) in [6] and Equation (2) and Equation (3) are derived from Equation (3) in [6] (see also Höhna (2013) Equation (2–4) [27]). These three probability functions are used to define the probability density of the observed reconstructed tree.

The probability density of all speciation events 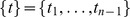 in the reconstructed tree under complete sampling (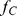) is
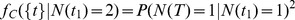


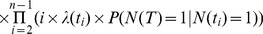
(4)which was derived by Thompson for constant rates (Equation (3.4.6) though with a different combinatorial factor which represents the probability of the tree topology)[28]. Additionally, I modified the likelihood to use the time-dependent rate functions of [6].

The probability density under diversified taxon sampling can be derived by recognizing that all speciation events are independent and identically distributed [2]. The definition of diversified sampling dictates that all unobserved speciation events leading to extant species occurred after the last observed speciation event. Hence, the probability of each unobserved speciation event is 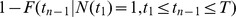 where 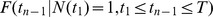 is the probability that a speciation event has occurred prior to 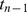, see Figure 2 for an illustration.

The probability density function of the divergence times for non-constant rates is (adopted from [27] Equation (8))
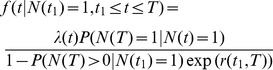
(5)and thus the distribution function being
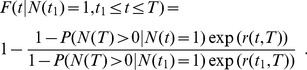
(6)


A short proof of Equation (6) is given in the Appendix.

From [2] Equation (A.1) in a slightly different notation we have the probability density function of the speciation events in the reconstructed tree given the time of the most recent common ancestor (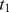) and the total number of species 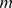 (sampled and missing species) at the present time
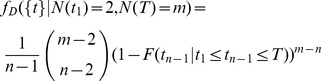


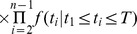


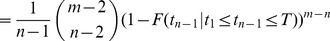


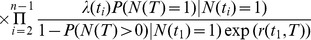
(7)


The probability density conditioned only on the time since the most recent common ancestor is obtained using Equation (12) in [27]

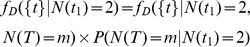


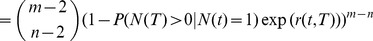


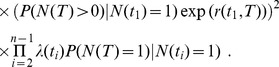
(8)


In the remainder of the paper I will use Equation (8) for the computation of the likelihood under diversified taxon sampling. Equation (4) with the rate function 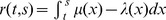 if 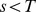 and 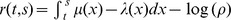 if 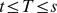 will be used for the likelihood function under uniform taxon sampling (see [6] Equation (31)). For the analysis I condition on starting with two species at the most recent common ancestor and on survival of the process.

### Parameters of the Birth-Death Process

I evaluated six different birth-death models with time-varying rates, proceeding from the simplest model to the most general and biologically realistic (Table 1). Other models with perhaps more complex patterns of rate variation over time are possible and could be of interest in future research. The first three models have been used previously but the last three models are new contributions.

**Table 1:** The six different birth-death models with the corresponding parameters.

Model	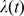	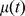
Model 1	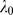	0
Model 2	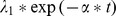	0
Model 3	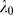	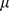
Model 4	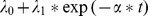	0
Model 5	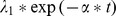	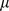
Model 6	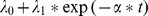	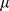

**Figure 3:**
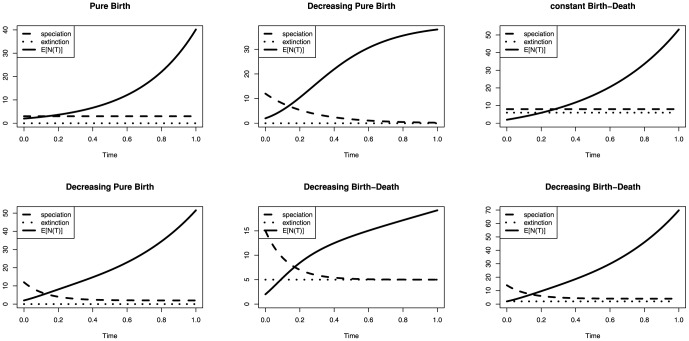
The six birth-death models used in this study. Each plot shows the speciation rate (dashed) and the extinction rate (dotted) over time, as well as the expected number of species (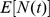) if the process endured for a time 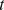. Model 1: A constant-rate pure birth process. Model 2: A decreasing pure birth process with the speciation rate declining towards zero as time continues. Model 3: A constant-rate birth-death process. Model 4: A pure birth process with a constant part of the speciation rate and a decreasing part of the speciation rate. Model 5: A birth-death process with an initial phase of expansion (higher speciation rate than extinction rate) and afterwards converging to a critical branching process. Model 6: A birth-death process with a constant extinction rate, a constant part of the speciation rate and a decreasing part of the speciation rate.

- Model 1: A constant-rate pure birth process, i.e. the Yule process [29]. The number of species is monotonically and exponentially increasing.
- Model 2: A decreasing pure birth process with the speciation rate declining towards zero. This process is equivalent to the decreasing rate pure birth process used in Rabosky and Lovette [14]. The number of species is monotonically increasing.
- Model 3: A constant-rate birth-death process, as used in Thompson [28]. The expected number of species increases exponentially with time.
- Model 4: A pure birth process with a decaying rate of speciation but a constant, non-zero speciation rate the longer the process continues (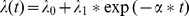). Thus, the process does not stop producing new species after the initial burst, as it is the case in Model 2. As for the other two pure birth processes, the number of species increases monotonically.
- Model 5: A birth-death process with an initial phase of expansion (higher speciation rate than extinction rate) and afterwards converging to a critical branching process, i.e. the speciation rate and the extinction rate are equal (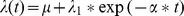 and 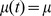). Although one might assume the expected number of species to remain constant for a critical branching process, this does not hold if the process is conditioned on survival (see Figure 3).
- Model 6: A birth-death process with a constant extinction rate, a constant part of the speciation rate and a decreasing part of the speciation rate. It represents the situation when diversity is continuously increasing starting with rapid radiation, followed by a steady expansion and some constant species turnover over time (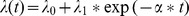 and 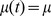).

The parametrization of the models can be found in Table 1. Figure 3 shows the expected number of species (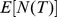) alive at time 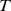. 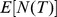 is obtained analytically using the fact that 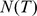 is geometrically distributed, see Equation (5) in [27]. Note that the process is conditioned on survival and thus 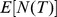 increases even if 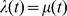.

### Accuracy of Parameter Estimation

Using simulation, I investigated the accuracy of parameter estimation under birth-death models – including pure birth models – and the impact of tree size on the accuracy. Additionally, I tested the effectiveness of the AIC [30] and BIC [31] for choosing between models. Finally, I applied the models on three empirical large-scale phylogenies.

The MLE is an unbiased estimator of the true parameters in the limit (if the number of observation goes to infinity) which has been shown using the Central Limit Theorem ([13], Chapter 5, page 118). Hence, for very large trees the MLE of the diversification rates is unbiased, and may or may not be biased for small samples. Condamine et al. [12] claimed that extinction rate estimates are unbiased if the inference model is the same as the simulation model and refer to [16] and [8]. I numerically investigated if the MLE is indeed unbiased if the model assumptions hold true. I simulated 1000 trees under complete taxon sampling for the time of the process 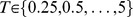 and conditioning on survival of the process under (1) a constant-rate pure birth process (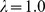) (2) a constant-rate birth-death process (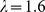, 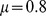) and (3) a birth-death process with a decreasing speciation rate (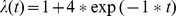, 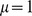). Then, I estimated the model parameters 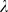, 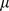 and 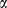 for each tree choosing the true model. These parameter settings were chosen so that the expected diversity after time 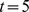 was approximately 200. Here, I varied the time of the process and thus also the size of the simulated tree to study the impact of the tree size on the bias of the estimated parameters.

I assumed a speciation rate larger than the extinction rate (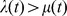). Hence the expected number of species in the phylogenetic tree (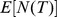) increases the longer the process takes. Therefore, I was able to observe if the bias decreases or increases with more taxa by running the birth-death process for different times. The simulations were performed in R using the package *TESS* [27].

### Model selection using the AIC and BIC

In frequentist inference the best model can be selected out of a set of (non-nested) candidate models via the AIC [30] and BIC [31]. The model with the lowest AIC (or BIC) score is selected as the best model. Here I used the AIC corrected (AICc) for small sample sizes,
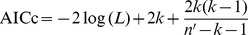
(9)where 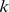 denotes the number of parameters in the model, 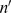 the number of speciation events (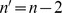) and 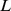 the likelihood score of the MLE [32], [33].

The AICc needs the number of observations (sample size) for the computation. One may ask what the sample size of one phylogenetic tree is: either just one single observation (i.e. one tree) or 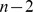 observations (the number of speciation events except the root). The choice will strongly influence the AICc score. I argue that a single phylogenetic tree represents a sample of size 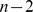. The number of species alive at time 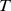 is geometrically distributed [6] and all speciation events in the reconstructed tree are independent and identically distributed (see [28] and [10]). Thus we have one observation from a geometric distribution and 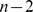 observation from some other distribution (Equation (6)) but the 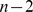 speciation events are dependent on the age of the tree and the number of sampled taxa. Therefore a single tree from a birth-death process resembles a sample of size 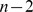 (see also [34]).

Rabosky [7] showed that the model selection by means of the AIC can be misleading. In Rabosky's study up to 50% of the simulations under a constant-rate birth-death process favored a more complex, non-constant rate model. Nevertheless, many analyses of birth-death models have been based on the ability of the AIC score to distinguish between models [14], [15]. Rabosky[17] used the AIC but Burnham and Anderson[32] showed that the AIC will favor more complex models for small sample sizes and the AICc should be used instead because it gives a higher penalty to more complex models. Similarly, the BIC is more conservative than the AIC. Therefore, I investigated the performance of the AICc and BIC to select the true model under which the data were simulated using the following setup.

I simulated 100 trees with 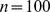 taxa under (1) a constant-rate pure birth process, (2) a decreasing-rate pure birth process and (3) a constant-rate birth-death process with 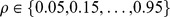 once under uniform taxon sampling and once under diversified taxon sampling applied to all three models. For the constant-rate pure birth process I choose the rate 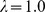; for the decreasing rate pure birth process I choose the rate function 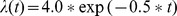 and for the constant-rate birth-death process I choose the rates 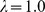 and 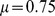. The tree size remained fixed for this study to resemble the size of empirical datasets. For each tree the best model out of the six mentioned models in Table 1 was selected.

### Analysis on Empirical Phylogenies

Sampling to maximize taxonomic representation is an acknowledged common practice in phylogenetic systematics (e.g. systematic studies that use a single species per family or genus [35]–[37]). However, the prevalence of diversified sampling and its importance in studies of lineage diversification are not well understood. I applied the birth-death models on three large-scale phylogenies of: ants, mammals and snakes. All three phylogenies are examples of diversified sampling.

Moreau et al. [24] built a phylogeny of extant ants sampled from nearly half of the described genera (139 of 288). The phylogeny contains 149 samples of the approximately 11,800 described species which gives a sampling probability of just above 1%. The estimated most recent common ancestor (MRCA) was 168 million years ago (Ma). Moreau et al. [24] did not estimate diversification rates but observed that most new lineages arose 100 to 70 Ma. I removed duplicate species per genus and the outgroup species to obtain a phylogeny with sampling as close as possible to diversified sampling.

Pyron and Burbrink [25] reconstructed a phylogeny of all known families and subfamilies of snakes. The phylogeny contains 41 samples of the nearly 3,500 snake species which again resulted into a sampling fraction of just above 1%. Pyron and Burbrink [25] studied clade-dependent diversification rates and found four diversification rate shifts. Furthermore, clade ages and diversification rates seemed to be negatively correlated.

Meredith et al. [4] constructed a family-level mammal phylogeny with 1–3 samples per family. The phylogeny contains 164 samples of the approximately 5,400 described species, a sampling fraction of approximately 3%. Their analysis revealed two rate increases, at 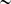
100 Ma and/or 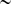
83 Ma, and a decrease at 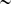
78 Ma. The original analysis only estimated rates until 40 Ma because of the sampling bias. I reduced the sample size so that exactly one species per family remained.

For each dataset the best model was selected by means of the AICc score and the BIC score. Each of the six models was applied under the assumption of uniform sampling and diversified sampling, giving a total of 12 different models. The empirical estimates of the sampling probability 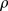 were used. The MLEs were obtained using the optimization routine *optim* in R repeated 10 times with different initial values to check for convergence. The R script and the phylogenies are available in the Dryad data repository at http://doi.org/10.5061/dryad.rd2s3.

#### Model Adequacy Testing

The adequacy of a model, often called model fit, is the probability of the observed data under the model [38]. Analytical computation of the probability of the data is infeasible in most situation and a simulation method, such as the parametric bootstrap, is necessary [39]. The parametric bootstrap computes the probability of the observed data (model adequacy) by simulating datasets, computing summary statistics for the simulated datasets and testing whether the summary statistic of the observed data falls within the 95% interval of the simulated summary statistics.

For each model and dataset I simulated 10000 trees under the MLE parameters conditioning on the time of the process equals the observed age of the tree and another 10000 trees under the MLE parameters conditioning on the number of taxa being equal to the number of observed (sampled) taxa. For the first 10000 trees I computed the empirical distribution of the 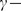
statistic [40] and the number of taxa sampled and for the second 10000 trees I computed the empirical distribution of the age of the trees. Finally I computed the probability of the summary statistics of the observed tree.

## Results and Discussion

### Accuracy of Parameter Estimation

**Figure 4:**
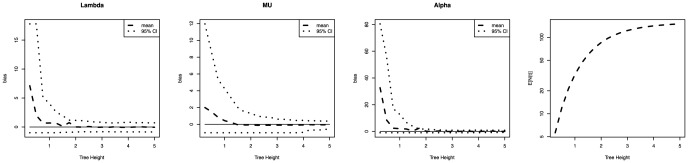
The bias in the maximum likelihood estimates of the speciation and extinction rate. The true parameters were 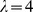, 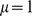 and 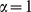. The figure shows that the bias decreases with larger trees (by simulating trees with a larger time 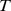). The expected number of species (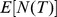) is presented to illustrate the increase in diversity over time.

Despite the claim of Condamine et al. [12] I observed in the simulation study that the MLEs were biased (see Figure 4) even under the true model. As expected, the bias decreased for larger trees and one may get unbiased estimates if the tree were very large. Even under a constant-rate pure birth process the MLE was biased for trees with fewer than 50 taxa compared with the results of Morlon et al. who found no bias, see Figure S4 in [8]. Specifically, the speciation rate was underestimated (see Figure S1 in File S1). However, under a constant-rate birth-death process the speciation rate was overestimated and the extinction rate underestimated (see Figure S2 in File S1).

Under the decreasing speciation rate birth-death model, the extinction rate 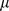 and the rate 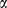 at which the speciation rate declines were also biased (see Figure 4). In these cases, 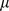 and 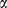 were overestimated. For small trees, the common observation of rapid radiations (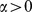, see[41]) could be due to the bias in the maximum likelihood estimates. Rabosky [7] observed this preference towards more complex models. Nevertheless, the parameters defining the time-dependent speciation and extinction rates of a birth-death process can be estimated with trees that are large enough.

Morlon et al. investigated if the estimated net-diversification rate (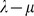) is biased, which is equivalent to my analysis under the constant-rate pure birth process. However, the bias is small for the simpler models, such as the constant-rate pure birth model. It is therefore conceivable that Morlon et al. attributed the bias to the rather large uncertainty in the parameter estimate (see the 95% confidence interval in Figure 4). Nevertheless, using extensive simulations the bias can be observed and reproduced and is increasingly important for more complex models.

Unfortunately the bias changes with different models and parameter values (see Figures S1 and S2 in File S1). A correction for the bias was not performed in this study because the empirical phylogenies are relatively large but could be important for smaller phylogenies or in future research. For a single study it is possible to simulate under the inferred parameter values and re-estimate the parameters in order to quantify the bias.

### Model Selection by means of AIC

**Figure 5:**
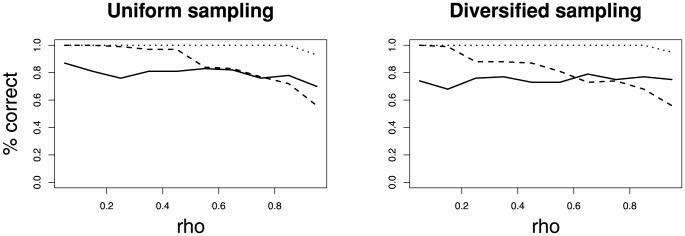
Sensitivity analysis of the success of the sample-size corrected Akaike Information Criterion to select the correct model. Trees were simulated under three different models: constant-rate pure birth (solid line), decreasing rate pure birth (dashed line) and constant-rate birth-death (dotted line). The x-axis shows simulations for different sampling probabilities 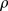.

My results indicate that the correct model can be inferred if the AICc score is used (see Figure 5). For the two constant-rate models the error rate did not depend on the sampling probability 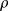. However, with increasing sampling probability the error rate increased for the decreasing rate pure birth model (Model 2).

These results are more optimistic than the results of [7]. The improvements are due to the advantage of the AICc over the AIC. Nonetheless the AICc is not flawless and the accuracy depends on the sample size and model complexity.

**Figure 6:**
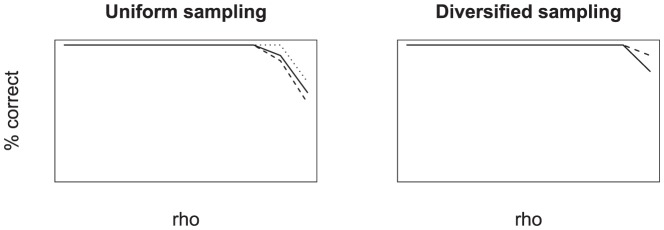
The sensitivity analysis testing whether the sampling strategy can be inferred. Trees were simulated under three different models: constant-rate pure birth (solid line), decreasing rate pure birth (dashed line) and constant-rate birth-death (dotted line). The x-axis shows simulations for different sampling probabilities 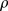.

Furthermore, I tested, using the same simulation scheme, if the correct sampling method could be inferred. The ability to identify the correct sampling strategy based on a likelihood method has not been investigated previously. In [2] we only investigated the effect of the sampling strategy on the inferred parameters. The results show that the sampling method can clearly be recovered (Figure 6). The error rate starts to increase sharply only for almost complete sampling. This is to be expected because uniform taxon sampling and diversified taxon sampling are identical for 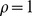 [2].

The same exercise was repeated using the BIC score for model selection. The results are very similar with the difference that the error rate of the constant-rate pure birth model was lower and the error rate of the decreasing rate pure model was higher (see Figure S3 and S4 in File S1). This result is not surprising because the BIC is more conservative and thus penalizes the more complex model (the decreasing rate pure birth model) more intensively.

### Empirical Analyses

**Figure 7:**
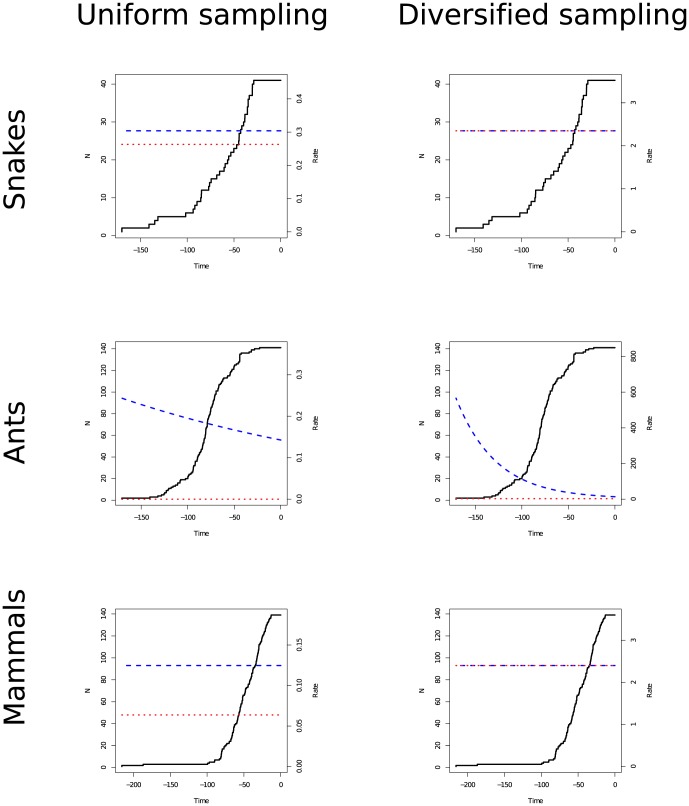
The estimated diversification rates for the snake, ant and mammal dataset. The plots show the change over time in total number of surviving lineages (solid black line), the speciation rate (dashed blue line) and the extinction rate (dotted red line) is given.

Diversified sampling was identified as the best-fitting model for the snake phylogeny. The best model was a constant-rate birth-death process with rates 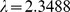 and 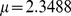, which resembles a critical branching process (see Figure 7). Note here that I conditioned on 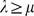 and with unconstrained parameters the extinction rate is estimated to be larger than the speciation rate. The difference in the AICc score between the best model under uniform sampling versus diversified sampling was 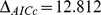, showing strong support for diversified sampling.

The inferred extinction rate being as high as the speciation rate (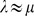) is surprising. Under a critical branching process, the net-diversification rate 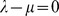 and the expected diversity remains constant. However, the snake clade accumulated as many as 3400 species in 169.7261 million years. The estimated extinction rate might thus be an artifact of the condition on survival of the process. Indeed, if the same inference procedure was repeated without conditioning on survival of the process, the extinction was estimated to be lower than the speciation rate (data not shown).

The mammal and the ant phylogenies favored a model with uniform sampling instead. The best model for the mammal phylogeny was the constant-rate birth-death model with rates 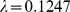 and 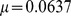. The best model for the ant phylogeny was a constant-rate pure birth process with 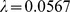, although a decreasing rate pure birth process gave a higher likelihood but not significant improvement (difference in likelihood scores: 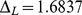).

Allowing non-constant diversification rates improved the model fit for the ant phylogeny but not for the mammal and snake phylogenies. The chosen time-dependent rate functions used in this study are likely too simplistic, as Meredith et al. [4] observed first an increase and then a decrease in speciation rates in the mammal phylogeny, a scenario that was not considered here. The derived likelihood equations can be used readily for other time-dependent rate functions. For example, it is possible to use the derived likelihood equations for a birth-death shift process with diversified taxon sampling [7], [9].

The parametric bootstrap analyses revealed that most models are not adequate for the observed data and it is very improbable (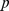-value smaller than 0.01) that the data were generated under these models (see Table S1–S3 in File S1). This results emphasizes that the models are too simplistic and some model assumptions are violated, e.g. a constant rate function. Only the constant-rate pure birth model could not be rejected according to its model fit for the ant phylogeny. Note that the constant-rate birth-death model was also not rejected but the estimated extinction rate was zero. However, the constant-rate pure birth model was not the best model according the AICc and BIC. Thus, the AICc and BIC may favor a model with a worse fit and a model adequacy test, as performed here, should always accompany a model selection procedure.

### Possible Model Violations

The mathematical description I used here of diversified taxon sampling is rather crude. It requires that all speciation events before a given time are included and all speciation events after this time are excluded (see Figure 2). A relaxation of this assumption may yield much better estimates. For instance, very old speciation events that define splits of families or other higher-order taxa have a higher probability to be included. Still, the probability of a recent speciation event to be included is unlikely to be zero and some old speciation events may occasionally be missed.

For example, Meredith et al. [4] reconstructed the mammal phylogenetic tree based on 1–3 species per family. Including at least one species per family is clearly an example of diversified taxon sampling, although not as strict as in the mathematical definition used here. The present strict definition of diversified sampling would require the missing species (more than 5200) to have speciated after the last sampled speciation event, which was 6.73 million years ago. Instead, the diversified sampling could be used as a process for higher-order phylogenies, as described by [42]. A suitable cut-off is needed after which all speciation events are discarded and then the diversified sampling can be applied. The advantage of the method in the present paper is that it includes non-constant diversification rates in contrast to the method in [42].

A simple test showed that assuming a broader time interval when the missing speciation events could have occurred improved the likelihood score enormously. I removed the last 20% of the speciation events because these are likely to violate the diversified sampling scheme. Diversified sampling was then preferred over uniform sampling on these pruned phylogenies (data not shown). Hence, diversified sampling is very sensitive to small violation of the sampling procedure. However, this data manipulation is treacherous and may mislead conclusions.

It seems promising to extend diversified sampling by softening the constraint that all missing speciation events must have occurred after the last observed speciation event. A possible extension is to allow speciation events to be sampled with probability 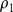 before some given time 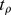 and with probability 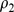 after this time. For example, we could have sample 80% of the speciation event before the last observed speciation event and 0% after the last speciation event. The diversified sampling method here is a special case of this extension when 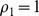, 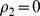 and 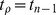.

## Conclusions

In the present paper I derived the probability density function for a birth-death process with non-constant rates and diversified taxon sampling. The birth-death process with diversified sampling allows biologists to analyze diversification rates of large-scale phylogenies that are sparsely sampled. I analyzed three such phylogenies including ants, mammals and snakes respectively.

The snake phylogeny supported the diversified taxon sampling model whereas the mammal and the ant phylogeny supported uniform taxon sampling. Nevertheless, it was obvious from the description of both the mammal and the ant study that diversified taxon sampling had been used but likely in a weaker sense than in the strict mathematical definition. The models presented in the current paper show improvements over the currently available models by allowing for non-constant rates and diversified sampling. Relaxing the mathematical definition of diversified taxon sampling used here and finding an intermediate between diversified taxon sampling and uniform taxon sampling appears to be the next step in future research.

In a simulation study I showed that the MLE of the diversification rates is biased, but if the trees are large enough the effect can be neglected. The AICc and the BIC can select the correct model and have a low false-negative rate. However, these results only apply under perfect condition when the true model is known and the model assumptions are not violated. Thus, it proved essential to investigate the fit of the models by means of parametric bootstraps. Here none of the models could provide a satisfactory fit when simulated trees were compared with the observed trees. Hence I recommend to simulate trees under the inferred parameters and compare the simulates tree with the observed trees instead of relying simply on the AICc and BIC to choose the best model.

The likelihood functions described in this paper are implemented in the R package *TESS* [27] and are freely available. The simulations of reconstructed trees were also performed with *TESS* and the R scripts for the model selection are available in the Dryad data repository at http://doi.org/10.5061/dryad.rd2s3.

## Supporting Information

File S1 **Derivation of the probability density function, additional simulation studies and tables of the analyses results.**
(PDF)
