# Supplementary material for: Likelihood Inference of Non-Constant Diversification Rates with Incomplete Taxon Sampling
Source: PLoS One. 2014 Jan 6;9(1):e84184. doi: 10.1371/journal.pone.0084184 (PMC3882215; doi:10.1371/journal.pone.0084184)
Supplement: File S1 — Derivation of the probability density function, additional simulation studies and tables of the analyses results. (PDF) [file pone.0084184.s001.pdf]

Supporting Information

Likelihood Inference of non-constant Diversification Rates with Incomplete Taxon Sampling

Running head: Birth-Death Process with Incomplete Taxon Sampling

Sebastian Höhna[1,*]

[1] *Department of Mathematics, Stockholm University, Stockholm, Sweden*

[*] *To whom correspondence should be addressed*

Keywords: Birth and death process, diversification, incomplete taxon sampling, Maximum Likelihood inference, Model assessment.

Sebastian Höhna
Department of Mathematics
Stockholm University
Stockholm
Sweden
E-mail: Sebastian.Hoehna@gmail.com

Derivation of the distribution function of a speciation time in the reconstructed tree

I will show that the derivative with respect to t of Equation (6) is identical to Equation (5):

$$\frac{d}{dt}\left(1-\frac{1-P(N(T)>0|N(t)=1)e^{(r(t,T))}}{1-P(N(T)>0|N(t_1)=1)e^{(r(t_1,T))}}\right) = \frac{\lambda(t)P(N(T)\!=\!1|N(t)\!=\!1)}{1-P(N(T)\!>\!0|N(t_1)\!=\!1)e^{(r(t_1,T))}}$$

The only factor depending on t is the second term in the denominator and the rest can be dropped

$$\begin{aligned}\frac{d}{dt}P(N(T)>0|N(t)=1)e^{(r(t,T))} &= \lambda(t)P(N(T)\!=\!1|N(t)\!=\!1)\\ \frac{d}{dt}\frac{e^{(r(t,T))}}{1+\int_t^T \mu(s)e^{r(t,s)}ds} &= \frac{\lambda(t)e^{r(t,T)}}{(1+\int_t^T \mu(s)e^{r(t,s)}ds)^2}\ .\end{aligned}$$

I use the rule that the derivative of $f(t)/g(t)$ equals $(f'(t)g(t)-f(t)g'(t))/g^2(t)$ with $f(t)=e^{r(t,T)}$, $g(t)=1+\int_t^T \mu(s)e^{r(t,s)}ds$ and the derivatives

$$\begin{aligned}\frac{d}{dt}f(t) &= \frac{d}{dt}e^{r(t,T)} = \frac{d}{dt}e^{\int_t^T \mu(s)-\lambda(s)ds} = \frac{d}{dt}e^{-\int_T^t \mu(s)-\lambda(s)ds}\\ &= -(\mu(t)-\lambda(t))e^{-\int_T^t \mu(s)-\lambda(s)ds} = -(\mu(t)-\lambda(t))e^{r(t,T)}\end{aligned}$$

and using Leibniz integral rule
$\frac{d}{dt}\left(\int_{a(t)}^{b(t)} f(t,y)\,dy\right) = f(t,b(t))\,b'(t) - f(t,a(t))\,a'(t) + \int_{a(t)}^{b(t)} f_t(t,y)\,dy$ with $a(t)=t$ and $b(t)=T$, thus

$$\begin{aligned}\frac{d}{dt}g(t) &= \frac{d}{dt}\left(1+\int_t^T \mu(s)e^{r(t,s)}ds\right)\\ &= 0-\mu(t)e^{r(t,t)}-(\mu(t)-\lambda(t))\int_t^T \mu(s)e^{r(t,s)}ds\\ &= -\mu(t)-(\mu(t)-\lambda(t))\int_t^T \mu(s)e^{r(t,s)}ds\end{aligned}$$

Finally, by combining the partial result and simplifying the equation I finish the proof

$$\frac{d}{dt}\left(1-\frac{1-P(N(T)>0|N(t)=1)e^{(r(t,T))}}{1-P(N(T)>0|N(t_1)=1)e^{(r(t_1,T))}}\right)$$
$$=\frac{1}{1-P(N(T)>0|N(t_1)=1)e^{(r(t_1,T))}}\times\frac{d}{dt}\left(\frac{e^{(r(t,T))}}{1+\int_t^T\mu(s)e^{r(t,s)}ds}\right)$$
$$=\frac{1}{1-P(N(T)>0|N(t_1)=1)e^{(r(t_1,T))}}$$
$$\times\frac{-(\mu(t)-\lambda(t)e^{r(t,T)})(1+\int_t^T\mu(s)e^{r(t,s)}ds)+(\mu(t)-\lambda(t))\int_t^T\mu(s)e^{r(t,s)}ds+\mu(t)e^{r(t,T)}}{(1+\int_t^T\mu(s)e^{r(t,s)}ds)^2}$$
$$=\frac{1}{1-P(N(T)>0|N(t_1)=1)e^{(r(t_1,T))}}\times\frac{\lambda(t)e^{r(t,T)}}{(1+\int_t^T\mu(s)e^{r(t,s)}ds)^2}$$
$$=\frac{\lambda(t)P(N(T)=1|N(t)=1)}{1-P(N(T)>0|N(t_1)=1)e^{(r(t_1,T))}}\qquad\blacksquare$$

Simulation study on the Maxim Likelihood Estimator

The aim of this simulation study is to identify the bias induced by the MLE on a constant rate pure birth model, a constant rate birth-death model and a decreasing speciation rate birth-death model. I simulated 1000 trees under complete taxon sampling for the time of the process $T \in \{0.25, 0.5, \ldots, 5\}$ and conditioning on survival of the process under (1) a constant rate pure birth process ($\lambda = 1.0$) (2) a constant rate birth-death process ($\lambda = 1.6$, $\mu = 0.8$) and (3) a birth-death process with a decreasing speciation rate ($\lambda(t) = 1 + 4 * \exp(-1 * t)$, $\mu = 1$). Then, I estimated the model parameters λ, μ and α for each tree choosing the true model. Here I present the results for the constant rate pure birth model and the constant rate birth-death model. The results of the birth-death model with a decreasing speciation rate was present in the

main text.

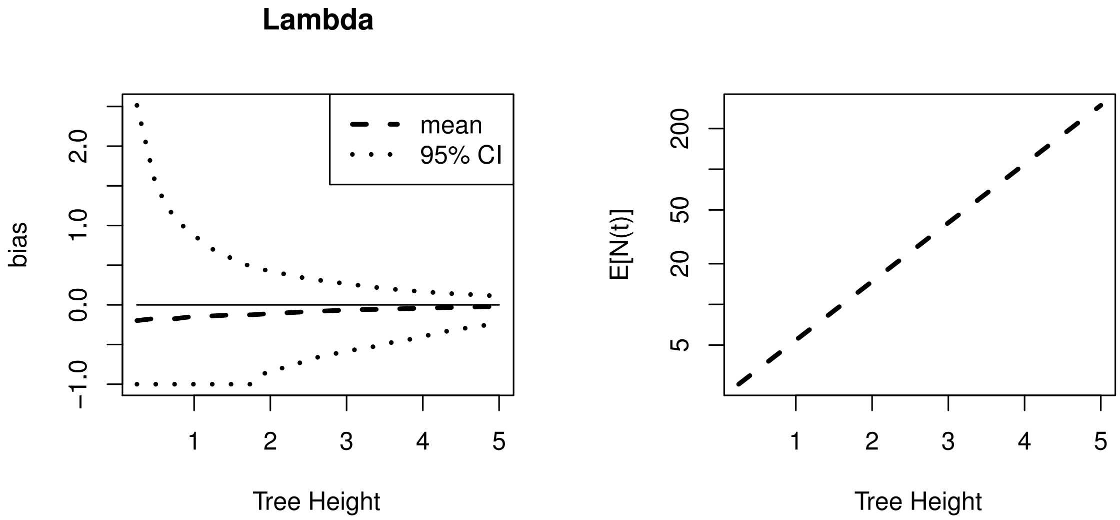

Figure S.1: The bias in the maximum likelihood estimates of parameters defining the speciation and extinction rate. The true parameters was $\lambda = 4$ and $\mu = 0$. The figure shows that the bias decreases with larger trees (by simulating trees with a larger time t). The expected number of species ($E[N(t)]$) is presented to illustrate the increase in diversity over time.

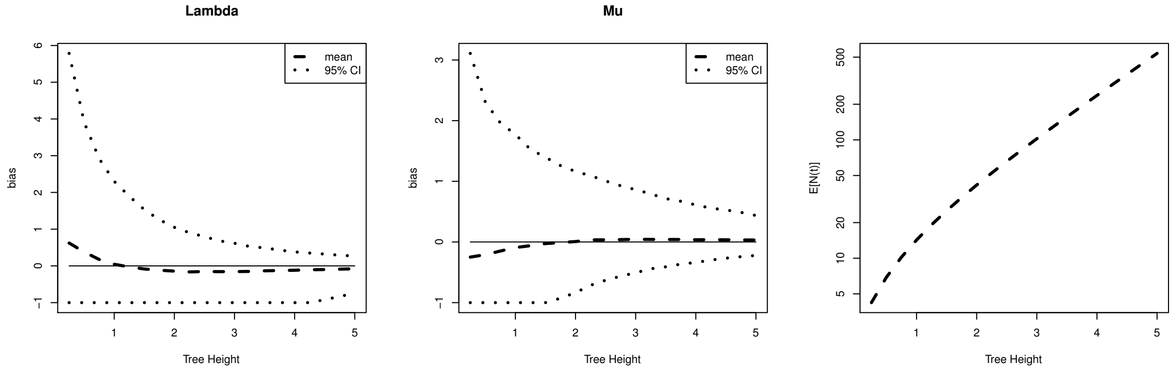

Figure S.2: The bias in the maximum likelihood estimates of parameters defining the speciation and extinction rate. The true parameters were $\lambda = 4$ and $\mu = 3.2$. The figure shows that the bias decreases with larger trees (by simulating trees with a larger time t). The expected number of species ($E[N(t)]$) is presented to illustrate the increase in diversity over time.

Simulation study on the Efficacy of the BIC

In the main text I discussed the efficacy of the Akaike's Information Criterion corrected for finite samples (AICc) to select the best model. Here, I repeat the simulation study but using the Bayesian Information Criterion (BIC) instead of the AICc. The simulation study design is as follows: I simulated 100 trees with $n = 100$ taxa under (1) a constant rate pure birth process, (2) a decreasing rate pure birth process and (3) a constant rate birth-death process with $\rho \in \{0.05, 0.15, \ldots, 0.95\}$ once under uniform taxon sampling and once under diversified taxon sampling. For each tree the best model out of the six mentioned models in Table 1 was selected. For the constant rate pure birth process I choose the rate $\lambda = 1.0$; for the decreasing rate pure birth process I choose the rate function $\lambda(t) = 4.0 * \exp(-0.5 * t)$ and for the constant rate birth-death process I choose the rates $\lambda = 1.0$ and $\mu = 0.75$.

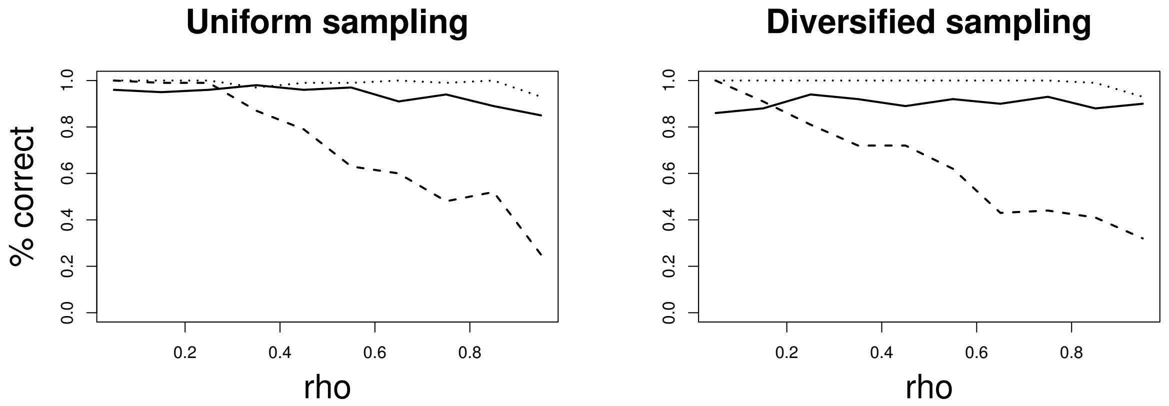

Figure S.3: Sensitivity analysis of the Bayesian Information Criterion to select the correct model. Trees were simulated under three different models: constant rate pure birth (solid line), decreasing rate pure birth (dashed line) and constant rate birth-death (dotted line). The x-axis shows simulations for different sampling probabilities ρ.

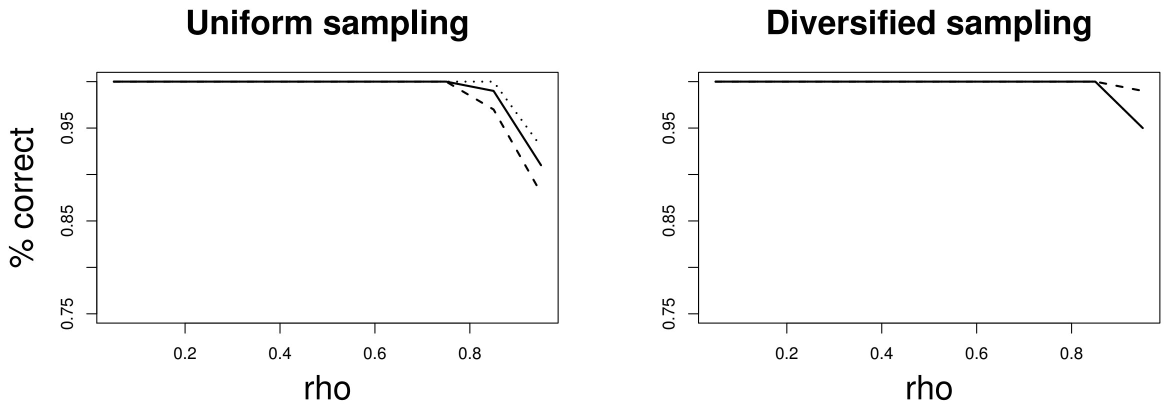

Figure S.4: The sensitivity analysis testing whether the sampling strategy can be inferred. Trees were simulated under three different models: constant rate pure birth (solid line), decreasing rate pure birth (dashed line) and constant rate birth-death (dotted line). The x-axis shows simulations for different sampling probabilities ρ.

Empirical results on the empirical phylogenies

I estimated the MLE for the six different models under uniform sampling and diversified sampling on three empirical datasets: ants [1], mammals [2] and snakes [3]. Here I present the results of the analyses including the model adequacy tests. The MLEs were obtained in R using the function *optim*, see also the R scripts deposited in the Dryad data repository at doi.org/10.5061/dryad.rd2s3. The model adequacy tests were performed using a parametric bootstrap by simulating 10000 trees under the MLE parameters and computing the number of taxa, the γ-statistic and the time of the process to reach n taxa.

Table S.1: Data set *Ants*

Model	log-Likelihood	AICc	BIC	p-value(gamma)	p-value(taxa)	p-value(treeheight)
1	-621.7704	1245.571	1248.454	0.746	0.115	0.533
2	-619.1779	**1242.446**	**1248.181**	0.991	0.006	1
3	-621.7704	1247.631	1253.366	0.738	0.142	0.538
4	-619.1779	1244.538	1253.094	0.991	0.006	1
5	-619.1779	1244.538	1253.094	0.992	0.006	1
6	-619.1779	1246.661	1258.006	0.99	0.005	1
7	-829.4413	1660.912	1663.795	0.01	0	0.999
8	-829.4413	1662.973	1668.708	1	0	1
9	-637.0724	1278.235	1283.97	0	0	1
10	-829.4413	1665.064	1673.621	1	0	1
11	-638.3722	1282.926	1291.482	0.751	0.128	0
12	-626.0846	1260.475	1271.82	1	0	1

Table S.2: Data set *Mammals*

Model	log-Likelihood	AICc	BIC	p-value(gamma)	p-value(taxa)	p-value(treeheight)
1	-642.524	1287.077	1289.982	1	0	1
2	-642.524	1289.136	1294.917	1	0	1
3	-640.0346	**1284.157**	**1289.938**	1	0	1
4	-642.524	1291.226	1299.851	1	0	1
5	-640.0346	1286.247	1294.873	1	0	1
6	-640.0346	1288.368	1299.807	1	0	1
7	-1367.745	2737.52	2740.425	0	0	1
8	-1367.745	2739.579	2745.36	1	0	1
9	-686.1957	1376.48	1382.26	0	0	1
10	-1367.745	2741.668	2750.294	1	0	1
11	-687.4036	1380.985	1389.611	0.46	0.024	0.009
12	-1202.169	2412.637	2424.076	1	0	1

Table S.3: Data set *Snakes*

Model	log-Likelihood	AICc	BIC	p-value(gamma)	p-value(taxa)	p-value(treeheight)
1	-202.6015	407.3056	408.9166	0.999	0.001	0.956
2	-202.6015	409.5188	412.6302	0.999	0.001	1
3	-196.4687	397.2532	400.3646	1	0	1
4	-202.6015	411.8517	416.3437	0.999	0.001	1
5	-196.4687	399.5861	404.0782	0.872	0.072	1
6	-196.4687	402.0486	407.7917	1	0	1
7	-306.2222	614.547	616.158	0	0	1
8	-306.2222	616.7602	619.8716	1	0	1
9	-187.8457	**380.0072**	**383.1186**	0	0	1
10	-306.2222	619.0931	623.5852	1	0	1
11	-190.0627	386.7741	391.2661	0.995	0.006	0.015
12	-187.357	383.8251	389.5683	1	0	1

References

[1] Moreau CS, Bell CD, Vila R, Archibald SB, Pierce NE (2006) Phylogeny of the ants: diversification in the age of angiosperms. Science 312: 101–104.

[2] Meredith R, Janečka J, Gatesy J, Ryder O, Fisher C, et al. (2011) Impacts of the Cretaceous Terrestrial Revolution and KPg extinction on mammal diversification. Science 334: 521–524.

[3] Pyron RA, Burbrink FT (2012) Extinction, ecological opportunity, and the origins of global snake diversity. Evolution 66: 163–178.
